# A Cost-per-Responder Analysis of Ritlecitinib vs Baricitinib in Severe Alopecia Areata

**DOI:** 10.36469/001c.159914

**Published:** 2026-06-11

**Authors:** Ashley S. Cha-Silva, Kate H. Zhang, Christopher N. Graham, Samantha K. Kurosky, Helen Tran, Ernest H. Law, Eingun J. Song

**Affiliations:** 1 Pfizer, Inc., New York, New York; 2 RTI Health Solutions, Research Triangle Park, North Carolina; 3 Frontier Dermatology, Mill Creek, Washington

**Keywords:** alopecia areata, cost-per-responder, ritlecitinib, baricitinib, treatment costs

## Abstract

**Background:**

Ritlecitinib, a JAK3/TEC family kinase inhibitor, and baricitinib, a JAK1/2 inhibitor, are approved for treating severe alopecia areata (AA).

**Objective:**

Develop a cost-per-responder analysis for ritlecitinib and baricitinib for treating severe AA to estimate the budget impact based on clinical efficacy, available dosages, and pricing structure.

**Methods:**

A decision tree evaluated cost implications of once-daily ritlecitinib 50 mg, or baricitinib 2 mg and 4 mg treatment over 1 year from a US perspective. Drug costs were estimated using wholesale acquisition costs. Distribution across treatment and dosing options were based on real-world evidence. We defined shorter-term response as Severity of Alopecia Tool (SALT) score relative change from baseline ≥30% (SALT Δ≥30%; SALT_30_) at Weeks 18 and 24, and longer-term response as absolute SALT score ≤20 at Weeks 36 and 52. Patients initiating baricitinib 2 mg with no shorter-term response could uptitrate to 4 mg or discontinue therapy. Decision probabilities were derived from real-world evidence and clinical trial data.

**Results:**

At Week 24, 52.10% of ritlecitinib initiators and 36.28% of baricitinib initiators achieved SALT Δ ≥30%; at Week 52, 40.26% and 30.63% of ritlecitinib and baricitinib initiators, respectively, achieved SALT ≤20. Baricitinib had a higher cost per responder than ritlecitinib at Weeks 24 (54 887vs45 577) and 52 (107 217vs94 834). Cost-equivalence was reached at Week 52 (38 180),with42.721532) and higher at Week 52 (difference, 10 728)thanonlybaricitinib2mguse,andloweratWeeks24and52(difference,−20 001 and −$60 073) than only baricitinib 4 mg use. The lower cost per responder for ritlecitinib 50 mg vs baricitinib 2 or 4 mg at Weeks 24 and 52, and greater early efficacy and fewer discontinuations is consistent with the results of a prior indirect treatment comparison that supported favorable outcomes with ritlecitinib 50 mg vs baricitinib 2 mg.

**Conclusions:**

Ritlecitinib 50 mg demonstrated a lower cost per responder than baricitinib 2 or 4 mg at Weeks 24 and 52, which may inform reimbursement or formulary inclusion of ritlecitinib for treating AA.

## INTRODUCTION

Alopecia areata (AA) is an autoimmune disease characterized by patchy or complete nonscarring hair loss on the scalp, with or without additional loss of facial and/or body hair.^1^ AA affects approximately 0.58% to 2% of the population worldwide^2–5^ and has an unpredictable and prolonged disease course, usually with persistent extensive hair loss.^6,7^ Additionally, patients with AA may experience psychological and psychosocial issues, such as anxiety and reduced participation in social activities, negatively impacting their quality of life.^8,9^

The Janus kinase (JAK) inhibitors deuruxolitinib, ritlecitinib, and baricitinib have been approved and are commercially available in the US for the treatment of severe AA.^10–12^ Ritlecitinib is a JAK3 and tyrosine kinase expressed in hepatocellular carcinoma (TEC) family kinase inhibitor approved for the treatment of both adults and adolescents aged 12 years and older with severe AA.^10^ Baricitinib and deuruxolitinib are JAK1/2 inhibitors approved for the treatment of adults (aged ≥18 years) with severe AA.^11,12^

Ritlecitinib 50 mg daily demonstrated efficacy in achieving scalp hair regrowth in the ALLEGRO phase 2b/3 study (NCT03732807) at 24 and 48 weeks.^13^ At Week 24, 23% of patients receiving ritlecitinib 50 mg daily achieved the primary endpoint of a Severity of Alopecia Tool (SALT) score of 20 or less (≥80% scalp hair coverage), with a significant difference in the response rate between the placebo and ritlecitinib 50 mg group (*P* < .0001). The proportion of patients in the ritlecitinib group with a SALT score of 20 or less continued to increase, with 43% of patients achieving a SALT score of 20 or less at Week 48.

The BRAVE-AA1/-AA2 trials (NCT03570749; NCT03899259) showed that baricitinib 2 mg and 4 mg were superior to placebo in achieving scalp hair regrowth.^14^ At Week 36, 38.8% of patients receiving 4 mg baricitinib, 22.8% receiving 2 mg baricitinib, and 6.2% receiving placebo in BRAVE-AA1 reached the primary endpoint of a SALT score 20 or less; 35.9%, 19.4%, and 3.3% had a SALT score 20 or less in BRAVE-AA2.^14^ At Week 52, 40.9% of patients receiving 4 mg baricitinib and 21.2% receiving 2 mg baricitinib in BRAVE-AA1 had a SALT score 20 or less; 36.8% and 24.4% had a SALT score 20 or less in BRAVE-AA2.^15^ Thus, a higher dose response was observed with baricitinib 4 mg than baricitinib 2 mg at 36 and 52 weeks.^14,15^

Despite the higher observed efficacy of the baricitinib 4 mg dose over the 2 mg dose, the label recommends starting with the baricitinib 2 mg dose once daily and increasing to the 4 mg dose if the response to treatment is not adequate.^11^ Payer coverage policies may also require initiation of the 2 mg dose given its lower cost. Ritlecitinib is currently available as a single 50 mg daily dose at a lower cost than baricitinib 4 mg daily, offering a straightforward dosing strategy.^16^

To the best of our knowledge, there has yet to be a cost-per-responder (CPR) analysis comparing ritlecitinib vs baricitinib for the treatment of severe AA. This study aimed to develop a CPR analysis for ritlecitinib 50 mg, and baricitinib 2 mg and 4 mg for the treatment of severe AA to estimate the budget impact of these new therapies resulting from differences in clinical efficacy, available dosages, and pricing structure.

## METHODS

The CPR model was developed using Microsoft Excel to evaluate the cost implications of once-daily ritlecitinib 50 mg, or once-daily baricitinib 2 mg and 4 mg treatment over a 1-year time horizon (52 weeks) from a US perspective. Patients entered the analysis receiving treatment for AA with ritlecitinib 50 mg daily (adult and adolescent [≥12 years] patients), baricitinib 2 mg daily (adults), or baricitinib 4 mg daily (adults). Drug costs were estimated using wholesale acquisition costs (**Supplementary Table S1**)^16^ at the drug level (ie, baricitinib 2 mg and 4 mg costs were combined based on the percentage of patients starting each dose) over a 1-year time horizon, using treatment continuation and response steps shown in the decision tree (**Supplementary Figure S1**).

The proportion of patients who first initiated baricitinib 2 mg vs 4 mg and subsequent dose change probabilities were extracted from an internal analysis of real-world electronic medical record data from the OMNY Health Base Data. The OMNY medical records are sourced largely from outpatient dermatology practices in the US and reflect real-world clinical decision-making for patients treated for dermatologic diseases. This analysis utilized data from January 2000 through July 2023 which included more than 53 000 patients with AA. Patients were selected from this cohort for analysis if they had at least 1 diagnosis code for AA and at least 1 valid prescription or administration of baricitinib. A total of 612 patients were eligible, of whom 67% initiated treatment with 2 mg and 33% with 4 mg, and 38% uptitrated from 2 mg to 4 mg.^17^

Treatment response was assessed at a shorter-term time point to determine whether the patient was responding to treatment, and at a longer-term time point to determine whether the treatment goal was reached. Shorter-term treatment response was defined as SALT score relative change from baseline of 30% or more (SALT Δ≥30% [SALT_30_]), and longer-term treatment response was defined as an absolute SALT score of 20 or less. The shorter-term and longer-term time points of response assessment were dependent on the data reported in the clinical trials for each treatment (ALLEGRO-2b/3 for ritlecitinib; BRAVE-AA1 and BRAVE-AA2 for baricitinib).^13,14^

Patients treated with ritlecitinib 50 mg had a shorter-term assessment of response (SALT Δ ≥30%) at Week 24. The longer-term assessment of response (absolute SALT score of ≤20) for ritlecitinib 50 mg was at Week 52, which was assumed to be equivalent to the final Week 48 assessment in the ALLEGRO-2b/3 trial.^13^ Patients receiving ritlecitinib or baricitinib with no response at the shorter- and longer-term assessments (except for patients receiving baricitinib 2 mg, who were uptitrated) were assumed to discontinue treatment. Patients treated with baricitinib 2 mg had a shorter-term assessment of response (SALT Δ ≥30%) at Week 18. Patients initiating baricitinib 2 mg who did not have shorter-term treatment response were allowed to uptitrate to 4 mg or discontinue therapy. All baricitinib 2 mg patients who uptitrated to baricitinib 4 mg were assumed to uptitrate at Week 18 based on real-world evidence (RWE), which indicated the mean (SD) time to a dose increase of baricitinib was 127 (79) days (~18 weeks).^17^ Uptitrated patients and patients who had a shorter-term response at Week 18 had a longer-term assessment of response (absolute SALT score of ≤20) at Week 36. The proportion of patients responding (absolute SALT score of ≤20) to baricitinib at Week 36 was obtained by digital extrapolation from SALT responder trajectories described in King et al^18^ using the TechDig software.^19^ Patients treated with baricitinib 4 mg had a shorter-term assessment of response (SALT Δ ≥30%) at Week 24, and a longer-term assessment of response (absolute SALT score of ≤20) at Week 52. The proportion of patients responding to baricitinib at Week 24 was obtained from the percentage change in SALT score from baseline and SALT responder trajectories described in King et al (2023).^18^ The proportion of patients responding to baricitinib at the shorter-term time points (SALT Δ ≥30%) was estimated based on the proportion of patients with an early (ie, by Week 12) or gradual (ie, during Weeks 12-36) response, as reported in King et al.^18^ Modeled baricitinib response at the shorter-term assessment point was estimated using all early responders and one-half of the gradual responders.

Decision probabilities were derived from RWE and clinical trial data (**Supplementary Table S2**).^17,18,20^ This analysis modeled pathways for populations aligned with the patients’ ages, per the US prescribing information (specifying ritlecitinib use for patients aged ≥12 years and baricitinib use for patients ≥18 years).^10^ The proportion of patients receiving baricitinib 2 mg and 4 mg and the proportion of baricitinib 2 mg nonresponders who uptitrated to baricitinib 4 mg were derived from a RWE study using the OMNY database (further described in the **Supplemental Material**).^17^ The rates of response to treatment for all drugs and respective doses were derived from the ALLEGRO-2b/3 trial for ritlecitinib,^13^ and from the BRAVE-AA1 and BRAVE-AA2 trials for baricitinib.^14^

The outcomes included in this analysis were as follows: (1) CPR, defined as the total drug costs up to a specific time point divided by the response at the same time point; (2) the proportion of baricitinib 2 mg patients who missed an opportunity for an effective dose, which was measured by the proportion of baricitinib patients who discontinued treatment after no response when there was an opportunity for an effective dose (ie, did not uptitrate); (3) the proportion of all baricitinib patients who missed an opportunity for an effective dose, defined as the proportion of all baricitinib patients who discontinued treatment after no response when there was an opportunity for a dose change; and (4) the proportion of baricitinib 2 mg patients who received an inadequate dose for 36 weeks, which was measured by the proportion of baricitinib 2 mg patients who did not respond at 36 weeks but achieved a response after uptitrating to baricitinib 4 mg.

Sensitivity analyses were conducted to assess the CPR of ritlecitinib 50 mg (adult and adolescent [≥12 years] patients), and baricitinib 2 mg and 4 mg (adult patients) in three scenarios: Scenario 1, baricitinib 2 mg only (all baricitinib patients received 2 mg); Scenario 2, baricitinib 4 mg only (all baricitinib patients received 4 mg); and Scenario 3, cost equivalence (the point when the percentage of patients initiating baricitinib 2 mg would result in the total baricitinib costs being equal to the total ritlecitinib costs) at Week 52. A scenario analysis was also conducted among adult patients only.

## RESULTS

### Base-Case

**Figure 1.**
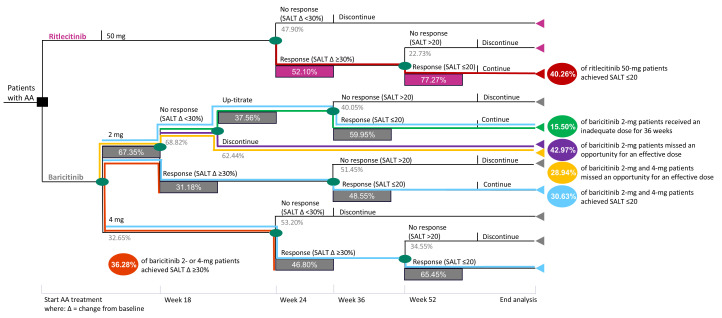
Decision-Tree Model Framework for SALT Response Probabilities for Baricitinib Dosing Schemes (Patients Aged ≥18 Years) and for Ritlecitinib 50 mg (Patients Aged ≥12 Years)^a^ Abbreviations: AA, alopecia areata; SALT, Severity of Alopecia Tool. ^a^The proportion of patients with response was calculated by multiplying probabilities along the same branch and adding probabilities along different branches. For example, the proportion of baricitinib 2 or 4 mg initiators who achieved SALT Δ ≥30% was calculated as follows: (32.65% × 46.80%) + (67.35% × 31.18%) = 36.8%

At Week 24, 52.10% of ritlecitinib 50 mg initiators and 36.28% of baricitinib 2 or 4 mg initiators achieved SALT Δ ≥30% (**Figure 1**). At Week 52, 40.26% of ritlecitinib 50 mg initiators and 30.63% of baricitinib 2 or 4 mg initiators achieved a SALT score of 20 or less. Baricitinib 2 or 4 mg had a higher CPR than ritlecitinib 50 mg at Weeks 24 ($54 887 vs $45 577) and 52 ($107 217 vs $94 834) (**Table 1**). Of the baricitinib 2 mg patients, 42.97% missed an opportunity for an effective dose (ie, those patients on baricitinib 2 mg who discontinued after no response to baricitinib 2 mg); 28.94% of baricitinib 2 mg and 4 mg patients missed an opportunity for an effective dose (ie, those patients on baricitinib 2 mg or 4 mg who discontinued after no response to baricitinib 2 mg); and 15.50% of baricitinib 2 mg patients received an inadequate dose for 36 weeks (ie, patients who responded to baricitinib 4 mg following up-titration after previously not responding to baricitinib 2 mg) (**Figure 1** and **Supplementary Table S3**). The results of the scenario analysis conducted among adult-only patients was consistent with those of the overall population. The decision-tree model and base-case results for the CPR model, including ritlecitinib 50 mg efficacy data from ALLEGRO-2b/3 for the adult population only, are provided in **Supplementary Figure S2** and **Supplementary Table S4**.

**Table 1.** Base-Case Results

	**Ritlecitinib 50 mg**	**Baricitinib 2 or 4 mg^a^**	**Absolute Difference^b^**
Total drug costs, $			
Week 24	23 746	19 911	3835
Week 52	38 180	32 841	5339
Responders, %^c^			
Week 24^d^	52.10	36.28	15.82
Week 52^d^	40.26	30.63	9.63
Cost per responder, $			
Week 24	45 577	54 887	–9310
Week 52	94 834	107 217	–12 383

^a^Represents the composite of all patients receiving baricitinib (2 mg and 4 mg).

^b^Ritlecitinib 50 mg is the reference group for the absolute difference.

^c^Shorter-term treatment response was defined as Severity of Alopecia Tool (SALT) score relative change from baseline ≥30% (SALT Δ ≥30%) at Weeks 18 and 24, and longer-term treatment response as absolute SALT score 20 or less at Weeks 36 and 52.

^d^The proportion of patients with response was calculated by multiplying probabilities along the same decision-tree branch and adding probabilities along different branches. For example, the proportion of baricitinib 2 or 4 mg initiators who achieved SALT Δ ≥30% was calculated as follows: (32.65% × 46.80%) + (67.35% × 31.18%) = 36.28%.

### Sensitivity Analysis

For the baricitinib 2 mg–only scenario (all baricitinib patients received 2 mg), ritlecitinib 50 mg CPR ($45 577) was lower than that for only baricitinib 2 mg ($47 109) at Week 24 (difference, −$1532) and higher at Week 52 (ritlecitinib 50 mg, $94 834; baricitinib 2 mg, $84 106; difference, $10 728) (**Supplementary Figure S3a**). For the baricitinib 4 mg–only scenario (all baricitinib patients received 4 mg), ritlecitinib 50 mg CPR ($45 577) was lower than that for only baricitinib 4 mg ($65 578) at Week 24 (difference, −$20 001) and Week 52 (ritlecitinib 50 mg, $94 834; baricitinib 4 mg, $154 907; difference, −$60 073) (**Supplementary Figure S3b**).

Cost equivalence was reached at Week 52 at $38 180, with 42.72% of baricitinib patients initiating 2 mg treatment. Ritlecitinib 50 mg CPR was $94 834 and baricitinib 2 or 4 mg CPR was $124 655 for total drug cost equivalence at 52 weeks (**Supplementary Figure S3c**).

## DISCUSSION

In this analysis of ritlecitinib and baricitinib for the treatment of severe AA, ritlecitinib 50 mg had a lower CPR than baricitinib 2 or 4 mg at Weeks 24 and 52. This was supported by the sensitivity analyses. The results of the ritlecitinib 50 mg in adults-only scenario were also consistent with the base case (ritlecitinib 50 mg in adults and adolescents). Although the total acquisition cost for ritlecitinib 50 mg at 24 and 52 weeks was higher than that for baricitinib 2 or 4 mg, ritlecitinib 50 mg resulted in a lower CPR due to higher response rates at an earlier stage and fewer discontinuations than baricitinib 2 mg. This is consistent with the results of an indirect treatment comparison that supported favorable outcomes with ritlecitinib 50 mg vs baricitinib 2 mg.^21^ Starting patients on a less effective dose (baricitinib 2 mg) may lead to treatment discontinuation in some patients, thus eliminating the opportunity for a response with a more effective dose. Receiving less-effective treatment can ultimately result in delayed optimal care.

Previous research has demonstrated a high economic burden of AA,^22–24^ supporting the need for cost-effective treatments. For instance, compared with the general population, patients with AA have higher total all-cause medical costs ($9154 vs $5787), largely due to higher inpatient costs, emergency department visits, prescription costs, and other costs.^23^ Furthermore, patients with alopecia totalis (total scalp hair loss) and/or alopecia universalis (total body hair loss) experience higher mean total all-cause medical and pharmacy costs than matched controls ($18 988 vs $11 030).^22^ Pharmacy ($1918 vs $487) and out-of-pocket costs ($2081 vs $751) are also higher for adolescent patients with alopecia totalis or alopecia universalis vs controls.^24^

To the best of our knowledge, there are no other published CPR analyses of baricitinib and ritlecitinib for the treatment of AA, which limits the interpretation of the findings in the context of existing literature. Nevertheless, an indirect treatment comparison found ritlecitinib 50 mg to have similar efficacy, based on the proportion of patients with SALT 20 or less and SALT 10 and less, to that of baricitinib 4 mg at Weeks 24 and 48 or 52.^21^ Therefore, given the similar efficacy observed across treatment doses, the lower CPR associated with ritlecitinib 50 mg compared with baricitinib 4 mg may inform discussions on the reimbursement of ritlecitinib 50 mg for the treatment of AA, acknowledging the uncertainty inherent in indirect comparisons and the potential influence of unmodeled factors.

In the indirect treatment comparison, the safety profile of ritlecitinib 50 mg was favored over 2 mg and 4 mg baricitinib in terms of serious adverse events and discontinuation due to adverse events, and baricitinib 4 mg had the highest rate of common adverse events.^21^ Nevertheless, safety considerations were not included in this analysis, and further safety comparisons between ritlecitinib and baricitinib are needed.

### Limitations

There are several limitations to this study. Given the modeling approach, the results may be sensitive to response assessment timepoints and up-titration timing. Short-term response was assessed earlier for baricitinib 2 mg (Week 18) based on results from a large US claims analysis, which indicated the average time to a baricitinib dose increase from baricitinib was 127 days.^17^ Although this may disadvantage baricitinib 2 mg by missing later responders (response at >18 weeks), this time point reflects real-world practice patterns in which disease management is at the clinician’s discretion, and typically accounts for additional weighing of overall benefit to risk and patient preference (eg, mitigation of prolonged exposure to less effective doses to balance treatment costs, time to response, and potential risks). These may vary across clinical practice. Long-term response was assessed at 52 weeks for both ritlecitinib 50 mg and baricitinib to align with clinical trial endpoint timing. Although the ALLEGRO-2b/3 trial evaluating the safety and efficacy of ritlecitinib 50 mg reported the primary efficacy endpoint (SALT ≤20) at 48 weeks and the BRAVE-AA1 and BRAVE-AA2 trials reported the baricitinib primary efficacy endpoint (SALT ≤20) at 52 weeks, a single time point was selected to reflect likely clinical practice where clinicians would evaluate patients at 1 year for response regardless of the specific product. Therefore the 52-week time point was selected and ritlecitinib 50 mg SALT ≤20 response at 48 weeks was assumed to remain stable at week 52. Given the known time-dependency of hair regrowth, the assumption of Week 48 outcomes from ALLEGRO 2b/3 representing the longer-term assessment at Week 52 is a conservative approach that may underestimate hair regrowth observed at the full 52 weeks.

Additionally, the model accounts only for drug acquisition costs, which do not include additional costs such as for laboratory tests, adverse events, or healthcare encounters associated with the treatment. The response values were based on naive comparisons, and head-to-head data between ritlecitinib and baricitinib are not available. Furthermore, real-world adherence was not fully accounted for due to lack of published analyses; however, discontinuations due to a lack of response were captured through the model assumptions. Given the lower efficacy of baricitinib 2 mg,^21^ clinicians are unlikely to recommend the use of only the 2 mg dose, thereby limiting the real-world applicability of Scenario 1. Finally, results beyond 1 year were not captured. As AA is a chronic condition that may require continued treatment to maintain response, future studies are needed to evaluate costs beyond the first year of treatment.

## CONCLUSIONS

In this analysis, ritlecitinib, currently available as a single dose of 50 mg daily, demonstrated a lower CPR than baricitinib 2 or 4 mg at Weeks 24 and 52. Thus, the simple and efficacious dosing strategy that ritlecitinib 50 mg offers may provide a better value for treatment response and potential budget optimization. These findings may inform discussions on reimbursement or formulary inclusion of ritlecitinib for the treatment of AA.

### Disclosures

Dr Tran, Dr Cha-Silva, Ms Kurosky, and Dr Law are employees of Pfizer Inc and own stock/stock options. Dr Zhang and Mr Graham are employees of RTI Health Solutions. Dr Song reports relationships with AbbVie, Acelyrin, Alphyn, Amgen, Arcutis, BMS, Boehringer Ingelheim, Dermavant, Eli Lilly, Galderma, Incyte, Janssen, Novartis, Ortho-Dermatologics, Pfizer, Sanofi & Regeneron, SUN, and UCB.

## Supplementary Material

Online Supplementary Material
