# Supplementary material for: A Cost-per-Responder Analysis of Ritlecitinib vs Baricitinib in Severe Alopecia Areata
Source: J Health Econ Outcomes Res. 2026 Jun 11;13(1):136–40. doi: 10.36469/001c.159914 (PMC13264044; doi:10.36469/001c.159914)
Supplement: Online Supplementary Material [file jheor_2026_13_1_159914_348872.pdf]

Journal of Health Economics and Outcomes Research

Online Supplementary Material

A Cost-per-Responder Analysis of Ritlecitinib vs Baricitinib in Severe Alopecia Areata. *JHEOR*. 2026;13(1):136-140. doi:10.36469/jheor.2026.159914

Table S1: Drug Cost Inputs

Table S2: Base-Case Data Sources

Table S3: Secondary Outcome Results

Table S4: Base-Case Results for Baricitinib 2 or 4 mg and Ritlecitinib 50 mg (Adult Population Only)

Figure S1: Decision-Tree Model Framework

Figure S2: Decision-Tree Model Framework for SALT Response Probabilities for Baricitinib Dosing Schemes and for Ritlecitinib 50 mg (Adult Population Only)

Figure S3: Cost per Responder for (a) Scenario 1 (Baricitinib 2 mg only [all Baricitinib Patients Received 2 mg]), (b) Scenario 2 (Baricitinib 4 mg Only [all Baricitinib Patients Received 4 mg]), and (c) Scenario 3 (Cost Equivalence at Week 52)

This supplementary material has been provided by the authors to give readers additional information about their work.

This is an open-access article distributed under the terms of the Creative Commons Attribution 4.0 International License (CCBY-4.0). View this license's legal deed at http://creativecommons.org/licenses/by/4.0 and legal code at http://creativecommons.org/licenses/by/4.0/legalcode for more information.

Supplementary Table S1. Drug cost inputs[16]

Drug	National Drug Code	Units/package	Package price, $	Cost/unit, $
**Ritlecitinib 50 mg**	00069-0334-28	28	3,957.69	141.35
**Baricitinib 2 mg**	00002-4182-30	30	2,739.99	91.33
**Baricitinib 4 mg**	00002-4479-30	30	5,479.98	182.67

Table S2. Base-case data sources

**Input**	**Base-case source**	**Decision probabilities**
Proportion of patients receiving baricitinib 2 mg and 4 mg	Real-world evidence study using the OMNY database[17]	Baricitinib 2 mg: 67.35%<br>Baricitinib 4 mg: 32.65%
Response to treatment for baricitinib 2 mg and 4 mg	Pivotal phase 3 BRAVE-AA1 and BRAVE-AA2 trials for baricitinib[14]	<u>Baricitinib 2 mg</u><br>Week 24 SALT Δ ≥30%: 31.18%<br>Week 52 SALT ≤20: 48.55%*<br><u>Baricitinib 4 mg</u><br>Week 24 SALT Δ ≥30%: 46.80%<br>Week 52 SALT ≤20: 65.45%*
Response to treatment for ritlecitinib 50 mg	Pivotal ALLEGRO-2b/3 trial for ritlecitinib[13]	Week 24 SALT Δ ≥30%: 52.10%<br>Week 52 SALT ≤20: 77.27%*
Proportion of baricitinib 2 mg non-responders who up-titrated to baricitinib 4 mg	Real-world evidence study using the OMNY database[17]	37.56%
Drug acquisition costs	Red Book, 2024[16]	See Table S1

* Of patients with response (SALT Δ ≥30%) at Week 24.

Table S3. Secondary outcome results

Outcome	Proportion of patients
**Proportion of baricitinib 2-mg patients who missed an opportunity for an effective dose**	42.97%
**Proportion of all baricitinib patients who missed an opportunity for an effective dose**	28.94%
**Proportion of baricitinib 2-mg patients who received an inadequate dose for 36 weeks**	15.50%

Table S4. Base-case results for the baricitinib 2 or 4 mg and ritlecitinib 50 mg (adult population only)

	Ritlecitinib 50 mg	Baricitinib 2 or 4 mg[a]	Absolute difference[b]
**Total drug costs, $**			
At Week 24	23,746	19,911	3,835
At Week 52	37,730	32,841	4,889
**Responders, %[c]**			
At Week 24[d]	50.48	36.28	14.20
At Week 52[d]	38.76	30.63	8.13
**Cost per responder, $**			
At Week 24	47,044	54,887	-7,843
At Week 52	97,346	107,217	-9,871

[a]Represents the composite of all patients receiving baricitinib (2 mg and 4 mg).
[b]Ritlecitinib 50 mg is the reference group for the absolute difference.
[c]Shorter-term treatment response was defined as Severity of Alopecia Tool (SALT) score relative change from baseline ≥30% (SALT Δ ≥30%) at Weeks 18 and 24, and longer-term treatment response as absolute SALT score ≤20 at Weeks 36 and 52.
[d]The proportion of patients with response was calculated by multiplying probabilities along the same decision tree branch and adding probabilities along different branches. For example, the proportion of baricitinib 2 or 4-mg initiators who achieved SALT Δ ≥30% was calculated as follows: (32.65% x 46.80%) + (67.35% x 31.18%) = 36.28%.

Figure S1. Decision-Tree Model Framework

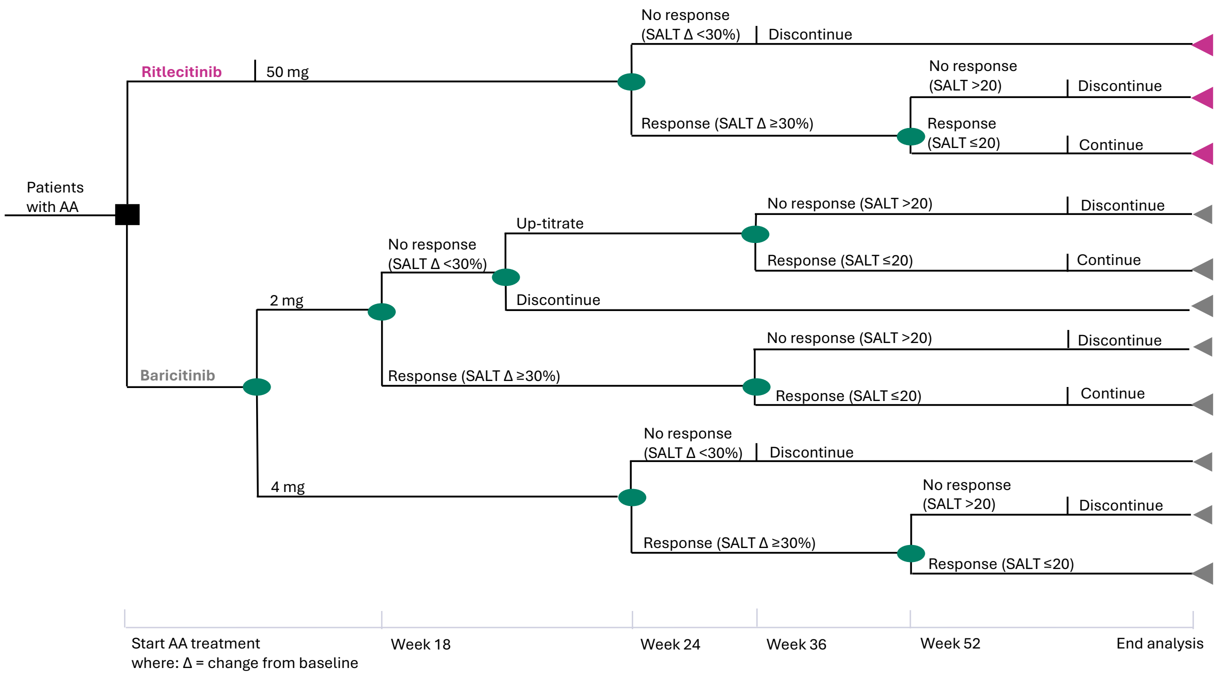

Abbreviations: AA, alopecia areata; SALT, Severity of Alopecia Tool.

Figure S2. Decision-Tree Model Framework for SALT Response Probabilities for Baricitinib Dosing Schemes and for Ritlecitinib 50 mg (Adult Population Only)

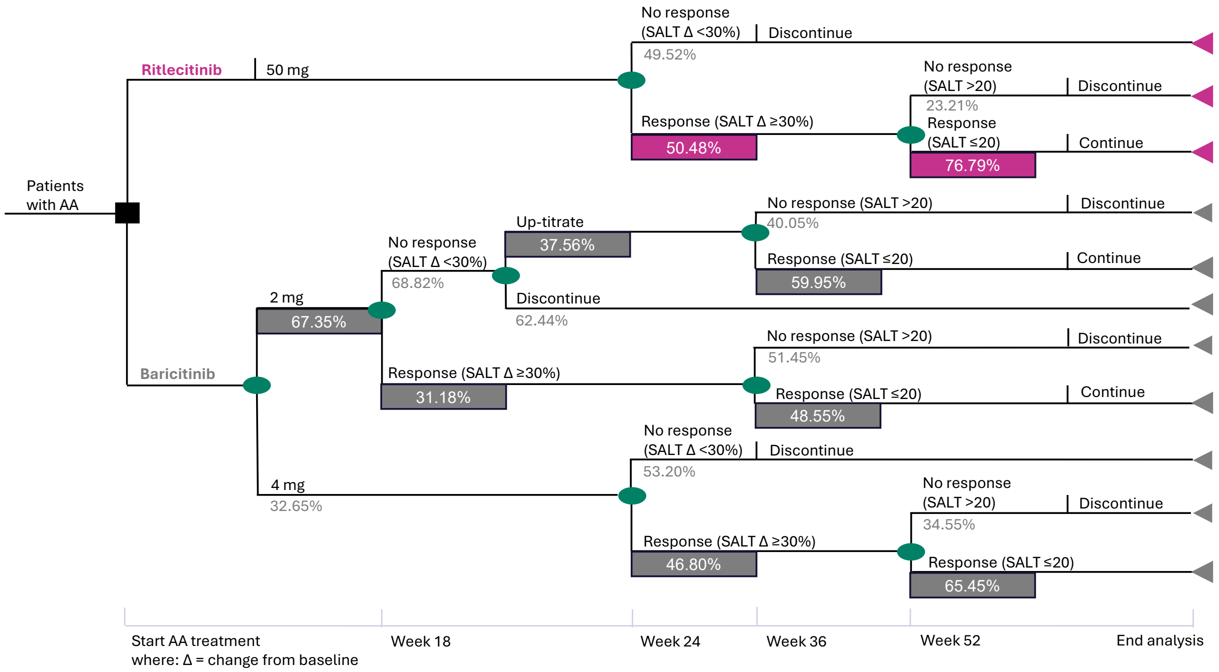

Abbreviations: AA, alopecia areata; SALT, Severity of Alopecia Tool.

Figure S3. Cost per Responder for (**a**) Scenario 1 (Baricitinib 2 mg only [all Baricitinib Patients Received 2 mg]), (**b**) Scenario 2 (Baricitinib 4 mg Only [all Baricitinib Patients Received 4 mg]), and (**c**) Scenario 3 (Cost Equivalence at Week 52)

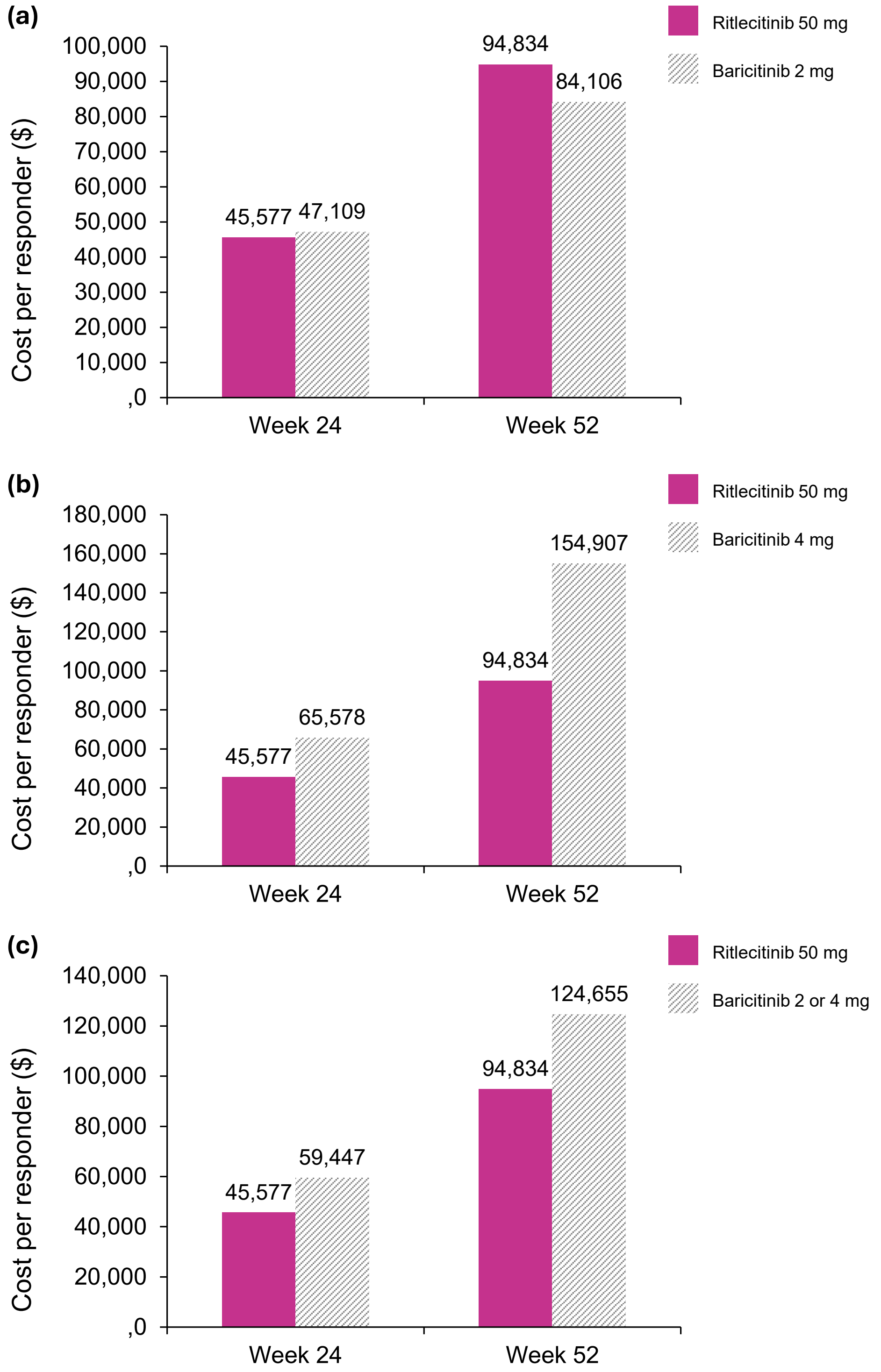

Shorter-term treatment response was defined as SALT score relative change from baseline ≥30% at Weeks 18 and 24, and longer-term treatment response as absolute SALT score ≤20 at Weeks 36 and 52.
SALT, Severity of Alopecia Tool.
